# High copy number and highly stable *Escherichia coli*–*Bacillus subtilis* shuttle plasmids based on pWB980

**DOI:** 10.1186/s12934-020-1296-5

**Published:** 2020-02-07

**Authors:** XingYa Zhao, JianYong Xu, Ming Tan, Jie Zhen, WenJu Shu, ShiBin Yang, YanHe Ma, HongChen Zheng, Hui Song

**Affiliations:** 1 University of Chinese Academy of Sciences, Beijing, 100049 China; 2 Industrial Enzymes National Engineering Laboratory, Tianjin Institute of Industrial Biotechnology, Chinese Academy of Sciences, No. 32 West 7th Avenue, Tianjin Airport Economic Area, Tianjin, 300308 China; 3 Tianjin Key Laboratory for Industrial Biological Systems and Bioprocessing Engineering, Tianjin Institute of Industrial Biotechnology, Chinese Academy of Sciences, Tianjin, 300308 China

**Keywords:** Expression vectors, pUC980, *Bacillus subtilis*, Alkaline pectate lyase, Alkaline protease, Pullulanase

## Abstract

**Background:**

pWB980 derived from pUB110 is a promising expression vector in *Bacillus* for its high copy number and high stability. However, the low transformation rate of recombinant plasmids to the wild cells limited the application of it. On the basis of pWB980, constructing an *E. coli*–*B. subtilis* shuttle plasmid could facilitate the transformation rate to *Bacillus* cells. Because the insertion site for *E. coli* replication origin sequence (*ori*) is not unique in pWB980, in order to investigate the best insertion site, eight shuttle plasmids (pUC980-1 ~ pUC980-8) containing all possible insertion sites and directions were constructed.

**Results:**

The results showed that all the selected insertion sites could be used to construct shuttle plasmid but some sites required a specific direction. And different insertion sites led to different properties of the shuttle plasmids. The best shuttle plasmids pUC980-1 and pUC980-2, which showed copies more than 450 per cell and segregational stabilities up to 98%, were selected for heterologous expressions of an alkaline pectate lyase gene *pelN*, an alkaline protease *spro1* and a pullulanase gene *pulA11*, respectively. The highest extracellular activities of PelN, Spro1 and PulA11 were up to 5200 U/mL, 21,537 U/mL and 504 U/mL correspondingly after 54 h, 60 h and 48 h fermentation in a 10 L fermentor. Notably, PelN and Spro1 showed remarkably higher yields in *Bacillus* than previous reports.

**Conclusion:**

The optimum *ori* insertion site was the upstream region of BA3-1 in pWB980 which resulted in shuttle plasmids with higher copy numbers and higher stabilities. The novel shuttle plasmids pUC980-1 and pUC980-2 will be promising expression vectors in *B. subtilis*. Moreover, the *ori* insertion mechanism revealed in this work could provide theoretical guidance for further studies of pWB980 and constructions of other shuttle plasmids.

## Background

*Bacillus subtilis* is a promising safe host strain for bio-industrial productions for its lack of pathogenicity and endotoxins [1–3]. Its capacity for secreting proteins directly into the medium facilitates the downstream processing greatly [4]. In the last 30 years, *B. subtilis* expression systems have been well developed. Many foreign proteins have been produced in *B. subtilis* cells successfully by using different kinds of expression plasmids [5–7]. However, compared to *Escherichia coli* expression system, relatively difficult in plasmid transformation, instability of plasmids, and lack of diversity for available plasmids became the main obstacles of the development of *B. subtilis* expression system [8, 9].

At present, the majority of available *B. subtilis* expression plasmids are derived from pC194 [10–12], pLS1 [13, 14] and pUB110 [3, 15]. In order to improve transformation efficiency of plasmids, a lot of *E. coli*–*B. subtilis* shuttle plasmids such as pHCMC05 [16] and pHT43 [17] have been constructed. However, the available plasmids in recent researches remain difficult to combine high copy number and high stability in the fermentation for producing heterologous proteins. As an example of high copy number plasmids, pWB980 [18] derives from plasmid pUB110 has been doing well in heterologous gene expression in *B. subtilis* [15, 19, 20]. However, the low transformation rate of the ligation products between pWB980 and target genes became a barrier to the widely use of it. On the purpose of solving this problem, many shuttle plasmids were constructed based on pWB980 [20, 21]. But so far, there is still a lack of systematical studies about the manipulations of pWB980.

In this work, the *ori* region where plasmid replication is originated in *E. coli* was inserted into four important sites of pWB980 to produce a series of *E. coli*–*B. subtilis* shuttle plasmids. Because the replication gene insertions may usually cause the changes of copy number and stability of the plasmids [22, 23], we focused on estimating the copy number and stability of the recombinant plasmids and found some promising *E. coli*–*B. subtilis* shuttle plasmids with high copy numbers and good stabilities. Moreover, an alkaline pectate lyase gene *pelN* from *Paenibacillus* sp. 0602 [24], an alkaline protease *spro1* from alkaliphilic *Bacillus* sp. 221 [25] and a pullulanase gene *pulA11* from *Anoxybacillus* sp. LM18-11 [26] were expressed more efficiently in the case of specific plasmids in *B. subtilis* WB600. Thus, we believe that these plasmids will be a wider range of uses in further research of the *B. subtilis* expression system.

## Results

### Construction of *E. coli*–*B. subtilis* shuttle plasmids

**Fig. 1:**
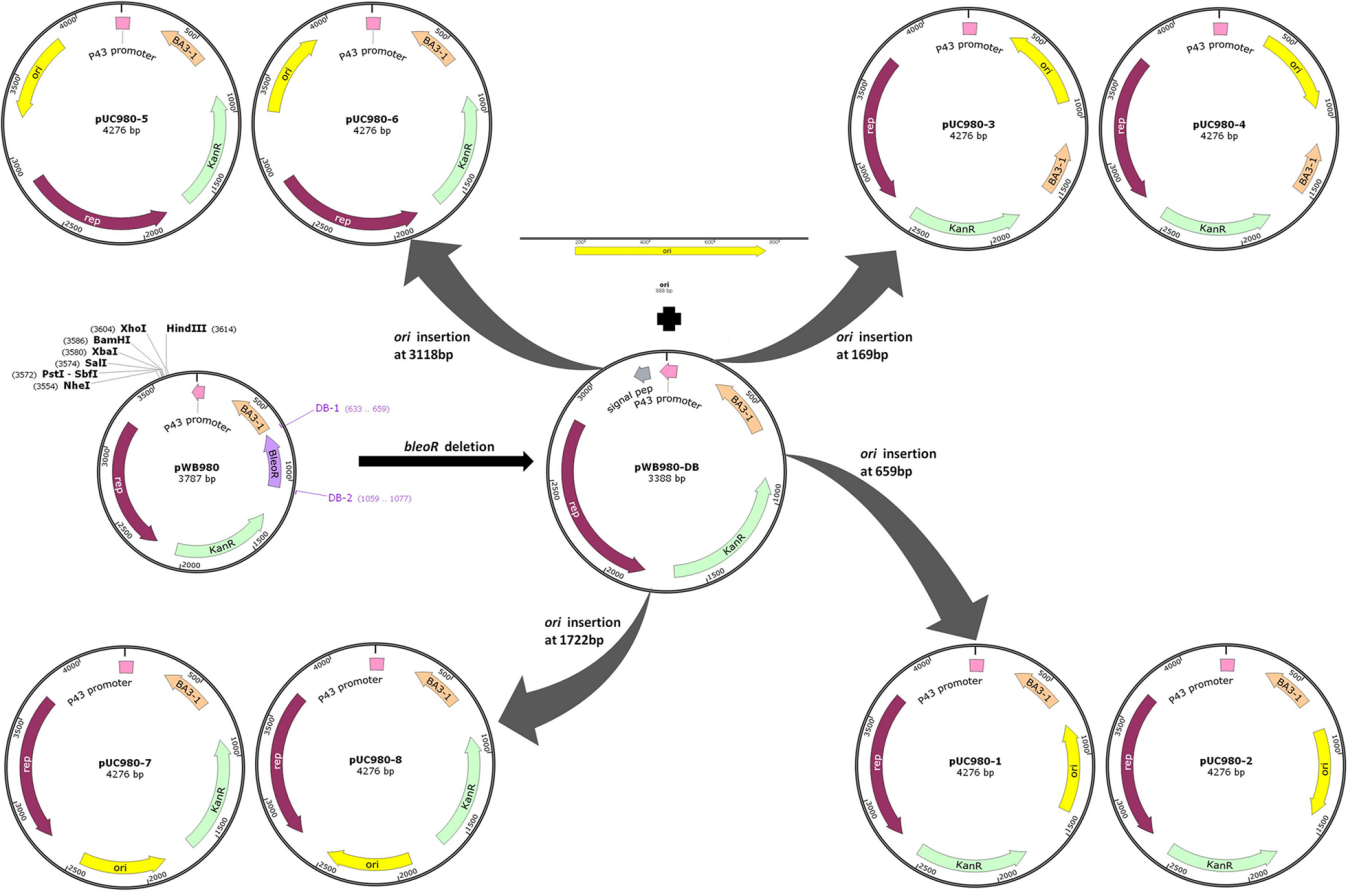
Plasmids constructions. The *bleoR* was deleted from pWB980, resulting plasmid pWB980-DB. It was integrated with the replication-originated region *ori* from pUC19 at four essential sites up- and down-stream the membrane-binding region BA3-1 and the replicase-coding gene *rep*

**Fig. 2:**
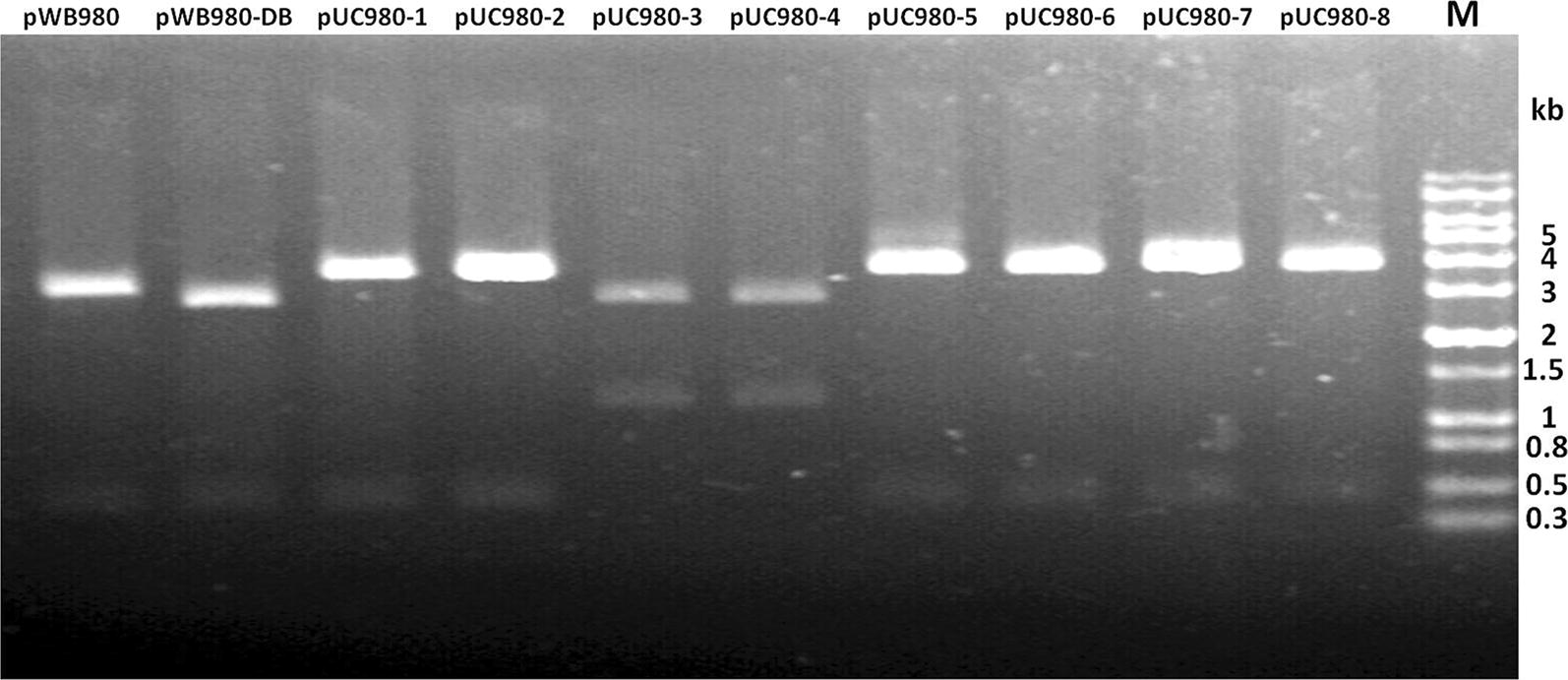
Verifications of constructed plasmids. Constructed plasmids were digested by *EcoR*I/*Hind*III restriction enzymes and separation by agarose gel electrophoresis. All plasmids were digested into two parts as shown in lanes. The lanes of pWB980 and pWB980-DB were 3337 bp, 450 bp and 2938 bp, 450 bp respectively. Lanes pUC980-1, pUC980-2, pUC980-5, pUC980-6, pUC980-7 and pUC980-8 were digested into the 3826 bp and 450 bp regions. Lanes pUC980-3 and pUC980-4 resulted into 2938 bp and 1338 bp regions. Lane M was DNA marker

**Fig. 3:**
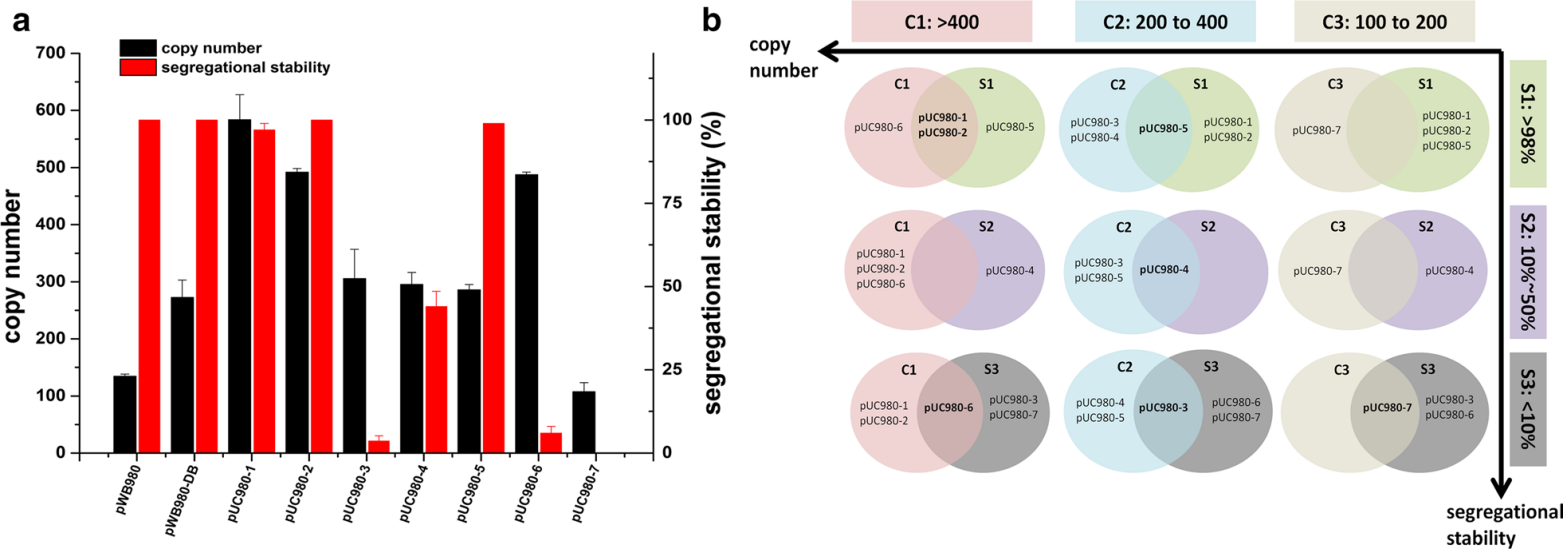
Plasmids copy number and segregational stability. **a** As shown in the graph, all the new plasmids obtained higher copy numbers than pWB980 except pUC980-7 and plasmid pUC980-8 was not counted because it was unreplicapble in *B. subtilis* cells. **b** plasmids were classified into three groups from high to low, concerning their copy numbers (C1 to C3) and segregational stabilities (S1 to S3)

In general, smaller size plasmids usually are easier to be manipulated. On the purpose of shortening pWB980 before construction, the more commonly used gene *kan* was kept while the *bleoR* was deleted from it (Fig. 1). As the selective gene *bleoR* was proved to be unnecessary in other studies and our previous work [19, 20]. The truncated plasmid pWB980-DB was successfully obtained in this work (Fig. 2). It was found to have the same stability with pWB980 along with a higher copy number (272) than that of pWB980 (134) as shown in the Fig. 3.

Then eight *E. coli*–*B. subtilis* shuttle plasmids derived from pWB980-DB were constructed successfully according to the design in Fig. 1. pWB980-DB was in the size of 3388 bp and the eight shuttle plasmids were all in the size of 4276 bp. To verify the plasmids clearly, double enzyme-digestions with EcoRI/HindIII were carried out and the results were shown in Fig. 2. All of them replicated normally both in *E. coli* and *B. subtilis* except pUC980-8 which could not replicate in *B. subtilis* host cells. This might be due to the interruption of reverse *ori* to the replication of the *rep* gene.

### Copy number of recombinant shuttle plasmids in *B. subtilis* 168

The copy numbers of all new plasmids were estimated and shown in the Fig. 3. Shuttle plasmids pUC980-1, pUC980-2 and pUC980-6 obtained high copy numbers of 584, 492 and 487 correspondingly. pUC980-1 had the highest copy number, which was over four times than that of pWB980 and twice than that of pWB980-DB. The copy numbers of plasmids pUC980-3 (305), pUC980-4 (295) and pUC980-5 (286) were similar to that of pWB980-DB. Only plasmid pUC980-7 with 107 copies per cell had a lower copy number than that of pWB980. pUC980-8 was not counted due to its un-replicability in *B. subtilis.* Therefore, the shuttle plasmids were classified into three groups (C1 ~ C3) according to their copy number levels from high to low as shown in Fig. 3b. Plasmids in C1 were determined to be high copy plasmids had copy numbers higher than 400, including pUC980-1, pUC980-2 and pUC980-6. C2 is the middle copy plasmids group included plasmids pUC980-3, pUC980-4, and pUC980-5 with copy numbers from 200 to 400. Plasmid pUC980-7 was ranked into C3 with relatively low copy numbers from 100 to 200.

### Stability of recombinant shuttle plasmids in *B. subtilis* 168

Plasmid stability consists of structural stability and segregation stability. All plasmids have exhibited structural stabilities after 30-days culturing and when digested with enzymes the expected fragments were obtained (Fig. 2). The plasmids were structurally stable and further verified by sequencing (data not shown). pWB980 and pWB980-DB were stable during passages with segregation stabilities of 100% (Fig. 3a). The plasmids from pUC980 series could be classified into three groups based on their segregation stabilities from high to low (Fig. 3b). Plasmids pUC980-1(99%), pUC980-2 (100%) and pUC980-5 (99%) were ranged into group S1 (high stable plasmid group). They acted well with more than 98% transformants retained after 30-days incubation in LB medium showing the similar stability with pWB980 and pWB980-DB. pUC980-4 were included in group S2 (low stable plasmid group) with a segregation stability of 39%. Plasmids in group S3 (unstable plasmids group) contained segregation stabilities lower than 10%, including pUC980-3 (2%), pUC980-6 (4%) and pUC980-7 (0).

Synthetically, all plasmids mentioned above were arranged and stated in Fig. 3b combining their ranks in copy number (C1 to C3) and segregation stability (S1 to S3). pUC980-1 and pUC980-2 were classified both in C1 and S1. Plasmid pUC980-6 in C1 obtained high copy number but low segregation stability in S3. Plasmid pUC980-5 in C2 were listed in S1, having relatively lower copy number than plasmids in C1. pUC980-4 performed moderately both in C2 and S2, while plasmid pUC980-3 in C2 was so unstable that ranked in S3. pUC980-7 was not only the most unstable plasmid but also had the lowest copy number, listed in C3 and S3. Based on the classification, plasmids pUC980-1 and pUC980-2 performing well both in copying and passaging were supposed to be the optimal expression vectors. Plasmids pUC980-5 and pUC980-6 only showed high stability and high copy number respectively. To further test the application potential of these new plasmids in production of industrial enzymes, three different industrial enzymes were selected for heterologous expression using these plasmids in *B. subtilis* WB600.

### Alkaline pectate lyase expression in *B. subtilis* WB600

**Fig. 4:**
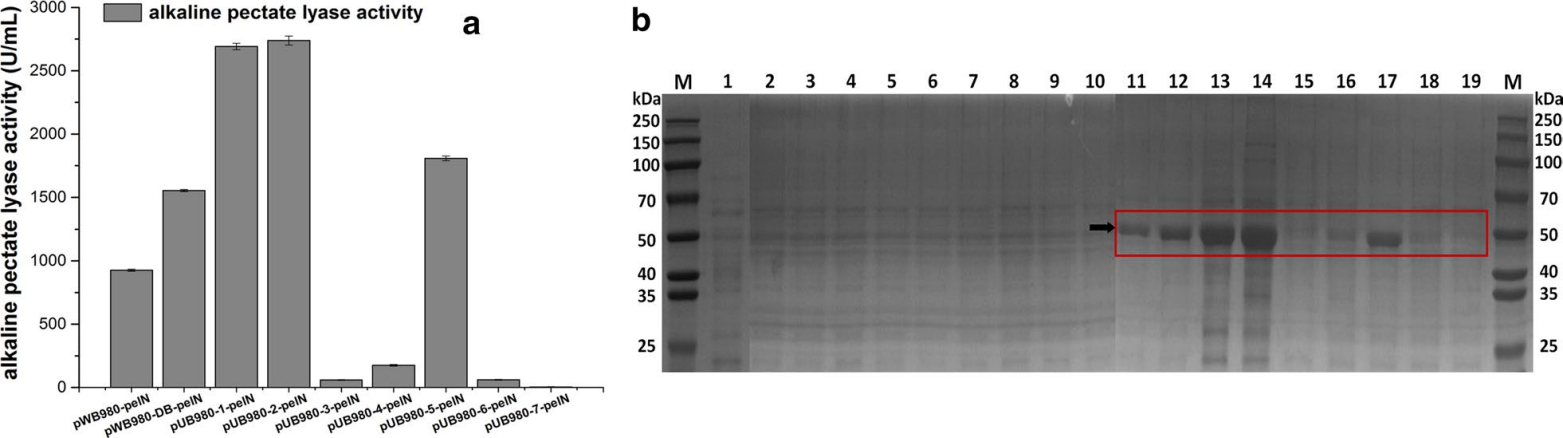
Alkaline pectate lyase productions in *B. subtilis*. **a** All plasmids were used to express alkaline pectate lyase *pelN* in *B. subtilis* WB600. The highest activity was obtained by pUC980-2 with 2738 U/mL. **b** SDS-PAGE analysis of PelN in culture supernatants. Lane M was protein molecular weight marker. The PelN protein was 48.0 kDa as shown in the frame. Lane 1 was the starting strain WB600, served as negative control. Lanes 2-10 were WB600 with plasmids pWB980, pWB980-DB and pUC980-serial plasmids (pUC980-1 to pUC980-7). Lanes 11-19 represented WB600 with PelN-expressing plasmids pWB980-*pelN*, pWB980-DB-*pelN* and pUC980-*pelN* plasmids (pUC980-1-*pelN* to pUC980-7-*pelN*) correspondingly

The alkaline pectate lyase gene *pelN* was expressed in the constructed plasmids. All plasmids functioned normally with extracellular secretory expressions after 48 h culturing in shake flasks. The results indicated obvious differences in expression levels as presented in Fig. 4a. The extracellular activities of PelN were more than 1500 U/mL when using pUC980-1, pUC980-2, pUC980-5 and pWB980-DB as expression vectors (Fig. 4a). The highest activity was 2738 U/mL with pUC980-2-*pelN*, which was nearly 3 times than that of pWB980. It also was the highest expression level in *Bacillus* [27, 28]. It is worth noting that the plasmids pUC980-3, pUC980-4, pUC980-6 produced activities lower than 200 U/mL. The lowest PelN activity was produced by pUC980-7 (4 U/mL) that was nearly the same to the background of the host cells.

In addition, comparative analysis of these expressions were carried through SDS-PAGE as shown in Fig. 4b. The SDS-PAGE analysis was performed and the intensity of observed bands correlated well with the PleN activities. The proportions of PelN protein in the supernatants at 48 h culturing with plasmids pWB980, pWB980-DB, pUC980-1 to pUC980-7 were estimated as 48%, 63%, 78%, 80%, 4%, 12%, 65%, 5% and 2% respectively (Fig. 4b).

### Alkaline protease expression in *B. subtilis* WB600

**Fig. 5:**
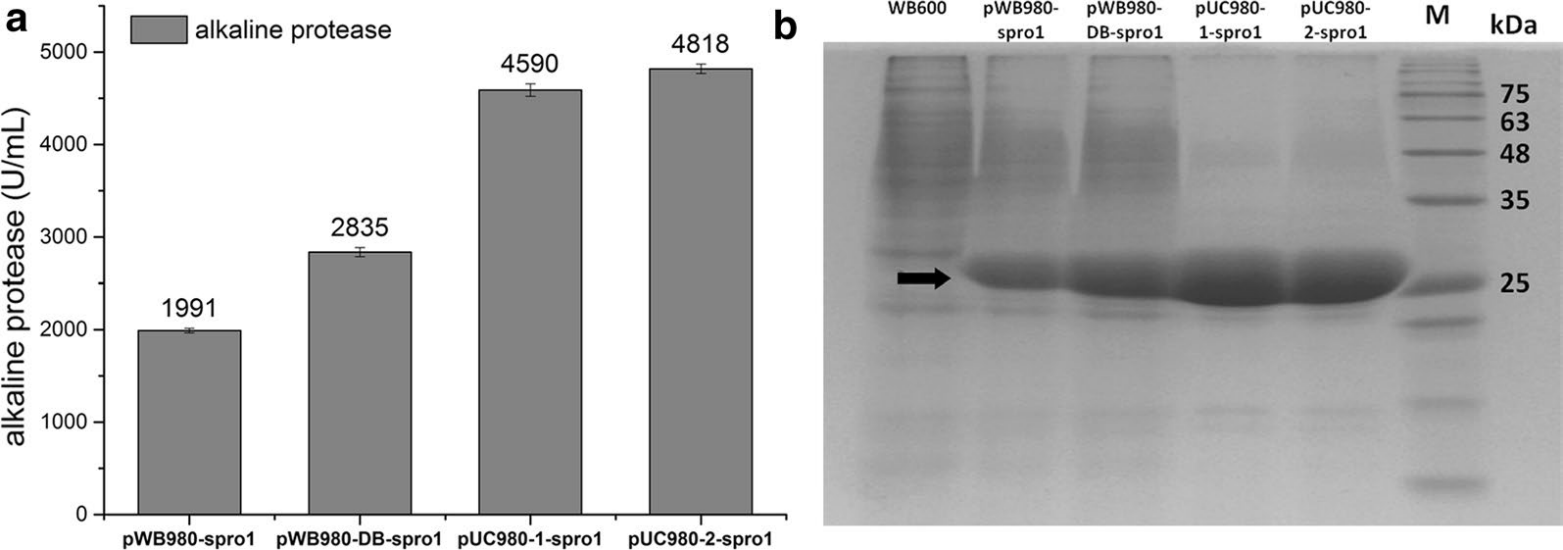
Alkaline protease productions in *B. subtilis* WB600. **a** Protease activities in supernatants were determined during Spro1-expressions with different plasmids in *B. subtilis* WB600. The highest activity was obtained by pUC980-2 with 4818 U/mL. **b** SDS-PAGE analysis of Spro1 in culture supernatants. Lane M was protein molecular weight marker. The Spro1 protein was 26.7 kDa as shown in the frame

As shown above, the pUC980-1 and pUC980-2 were proved to be useful in over-expressions of heterologous proteins. They were further used to express alkaline protease gene *spro1* in *B. subtilis* WB600, employing pWB980 and pWB980-DB as controls. After culturing in shake-flask for 48 h, the extracellular activities of protease expressed with pUC980-1-*spro1* and pUC980-2-*spro1* were 4590 U/mL and 4818 U/mL (Fig. 5a), which were 2.3 times and 2.4 times than that with pWB980-*spro1* (1991 U/mL), respectively. The proportions of the Spro1 in the supernatants were 72%, 77%, 92% and 95% in which expressed with plasmids pWB980, pWB980-DB, pUC980-1 and pUC980-2, respectively (Fig. 5b).

### Pullulanase expression in *B. subtilis* WB600

**Fig. 6:**
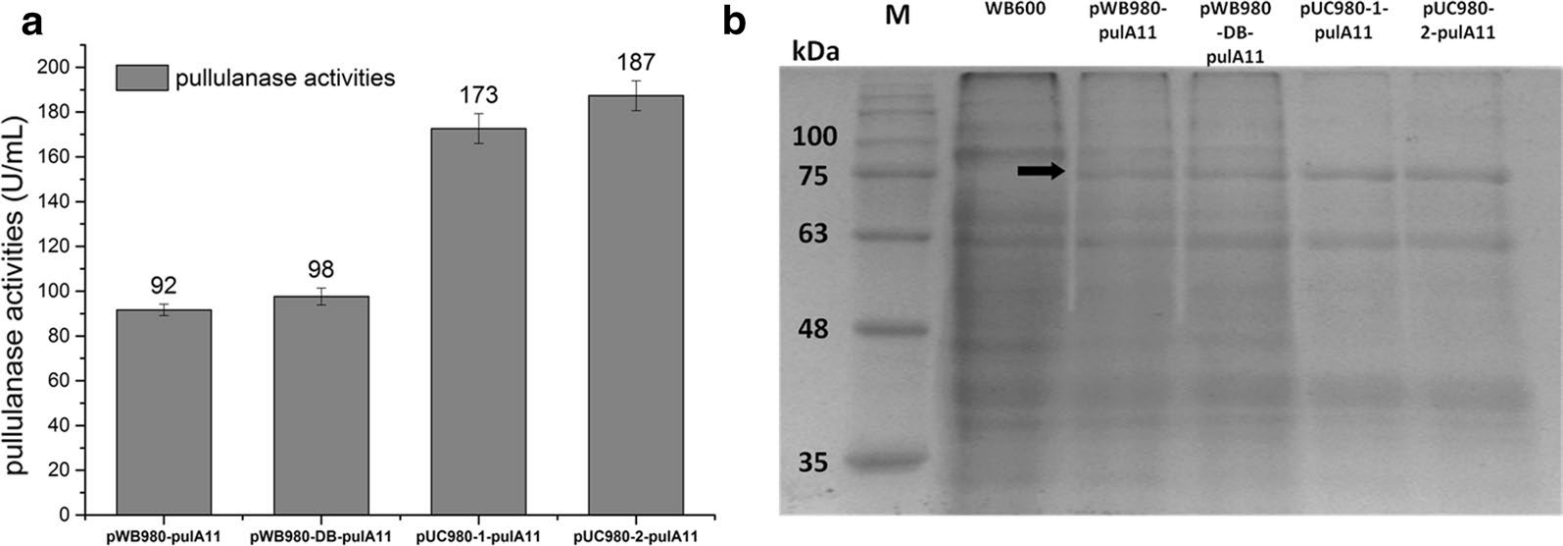
Pullulanase productions in *B. subtilis* WB600. **a** Activities of pullulanase expressed by different plasmids were determined in supernatants of WB600. The highest activity was obtained by pUC980-2 with 187 U/mL. **b** SDS-PAGE analyses of pullulanase expressions, M was the protein molecular weight marker. The PulA11 protein was 81.7 kDa as shown in the frame

Meanwhile, plasmids pUC980-1 and pUC980-2 were used to express a pullulanase gene *pulA11* in WB600 in shake flask culturing for 48 h. Enzyme activities of plasmids pUC980-1-*pulA11* and pUC980-2-*pulA11* in the supernatants were 173 U/mL and 187 U/mL respectively, which were 1.9 times and twice than pWB980 (92 U/mL) correspondingly as stated in Fig. 6a. The percentages of PulA11 proteins in the supernatants after 48 h fermentation of the recombinant strains containing plasmids pWB980-*pulA11*, pWB980-DB-*pulA11*, pUC980-1-*pulA11*, and pUC980-2-*pulA11* were 6%, 8%, 27% and 30%, respectively (Fig. 6b).

### Scale up fermentation of the optimum recombinant strains in a 10 L fermenter

**Fig. 7:**
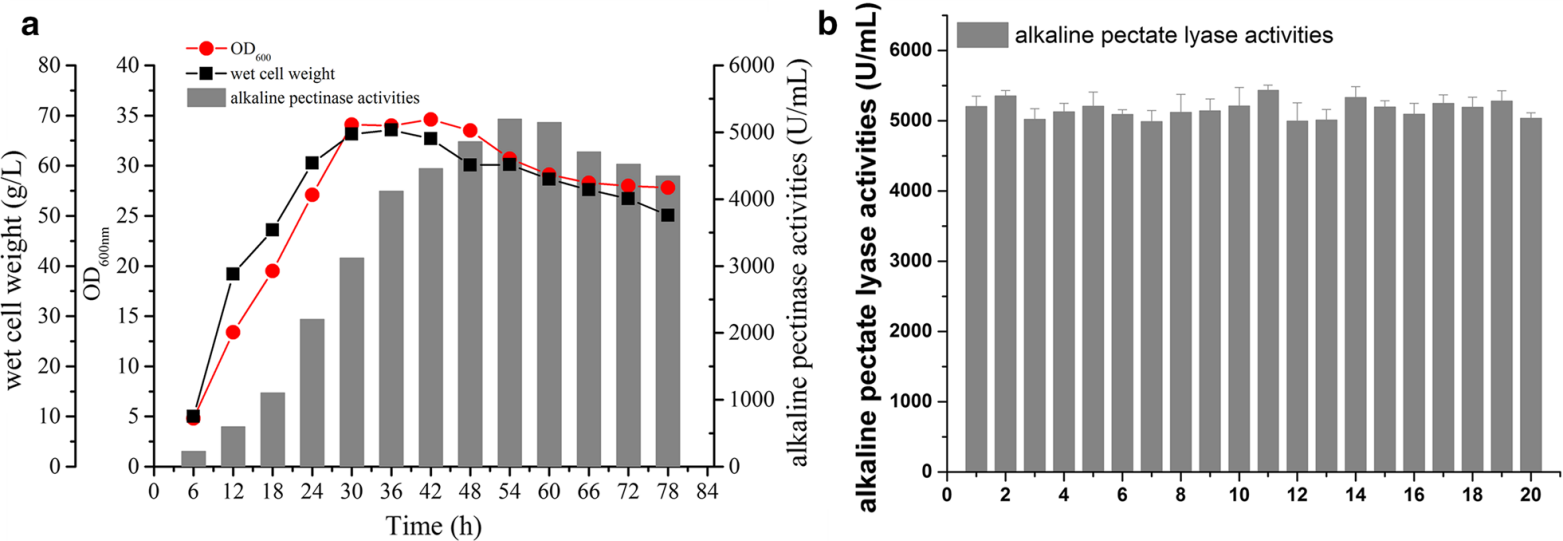
The 10 L fermentations of WB600 (pUC980-2-*pelN*). **a** The cell wet-weights, OD_600nm_ and the pectate lyase activities were determined for every 6 h. Cells went into the stationary phase after 30-hours fermentation and the cell wet-weight was 66.3 g/mL with OD_600nm_ of 34.1. The highest activity were obtained at 54 h with 5200 U/mL. Then the enzyme productions decreased gradually. **b** The fermentations of WB600 (pUC980-2-*pelN*) have been repeated for 20 times and the PelN activities kept stable and repeatable

The above results indicated that the recombinant strains containing pUC980-2-*pelN*, pUC980-2-*spro1*, and pUC980-2- *pulA11* showed the highest expression level in shake flask culturing basing on the remarkable properties of pUC980-2. In order to verify the stable production capacity of these strains in scale up fermentation, we performed batch-feed fermentations in a 10 L fermenter. The highest APL activity reached to 5200 U/mL after 54 h fermentation of the recombinant strain *B. subtilis* WB600 (pUC980-2-*pelN*). The curves for cell-growth and wet weight were shown in Fig. 7a. The cells grown into the stationary phase at 30 h with OD_600nm_ in 34.1 and 66.3 g/L of wet cell weight. The fermentation of this strain in a 10 L fermenter were repeated 20 times and the activities fluctuated little as shown in Fig. 7b. Furthermore, the highest production of Spro1 was 21,537 U/mL at 60 h and the highest production of pullulanase was 504 U/mL at 48 h (Additional file 1: Fig S1). So far, the extracellular yields of PelN and Spro1 by the recombinant strains *B. subtilis* WB600 (pUC980-2-*pelN*) and *B. subtilis* WB600 (pUC980-2-*spro1*) showed the highest level in *Bacillus subtilis* [29, 30].

## Discussion

The properties of pUB110-derived plasmid pWB980 were regulated by the *rep* gene and membrane-biding region BA3-1 according to previous studies [31–34]. The *rep* gene, encoding the replicase protein, was kept unchanged to fully support functioning of the pWB980 plasmid [18, 35–37]. The BA3-1 region was related to plasmid partitioning into the daughter cells [31, 38–40]. It has long been suspected that plasmid replication origin *ori* might be important for plasmids replications and partitioning [41, 42]. On these basis, the *rep* gene and the membrane-biding region have been manipulated with *ori* insertion separately.

Before *ori* insertions, the plasmid size was reduced for easier manipulation. The occasionally used resistance gene *bleoR* was deleted from pWB980, resulting plasmid pWB980-DB with a few higher copy numbers than pWB980 (Fig. 3a). The improvement of plasmid replication might be due to the elimination of the *bleoR* gene near the initial signal in BA3-1 region for lagging strand synthesis. Using pWB980-DB as the original plasmids, *ori* was inserted into four positions upstream and downstream the *rep* gene and the BA3-1 region.

As for shuttle plasmids derived from pWB980-DB, *ori* insertions at certain sites influenced the properties of each shuttle plasmid differently. Firstly, all *ori* insertions flanking the BA3-1 improved the plasmids copies in host cells concluded from the enhancements of copy numbers of plasmids pUC980-1, pUC980-2, pUC980-3 and pUC980-4. Moreover, insertions downstream BA3-1 affected plasmid segregations much more than the upstream ones and depressed plasmid stabilities sharply. These depressions strongly suggested the importance of this region to the distribution of plasmids in the cell division. In addition, *ori* insertions upstream the *rep* gene in plasmids pUC980-5 and pUC980-6 were conducive to plasmid replication. While there was a direction bias in plasmid segregations as the forward insertion was largely more stable than the reverse one. Furthermore, insertions of the *ori* downstream the *rep* gene resulted in poor performances in copy number and stability of pUC980-7 and pUC980-8 plasmids.

All plasmids (pUC980-1 ~ pUC980-7) kept structural stabilities in *B. subtilis*. They were systematically classified based on their copy numbers and segregation stabilities and further used to produce heterogenous proteins in *B. subtilis* WB600. Plasmids with good segregation stabilities dramatically outperformed the unstable ones, stated by the disparity between S1 plasmids and others. Moreover, the production of heterogenous proteins increased along with the copy numbers when the plasmids had similar stabilities. This conclusion was approved by the gradually improved expressions along the copy numbers of plasmids in group S1. And it was also supported by the plasmids in group S3. As for plasmids with similar copies, improvements in segregation stabilities enhanced the productions of heterogenous proteins notably, corroborated by plasmids in every segregation stability group.

Plasmids pUC980-1 and pUC980-2 performed well in expressing the tested heterogenous proteins, especially plasmid pUC980-2, with which the highest productions of alkaline pectate lyase PelN and alkaline protease Spro1 were obtained in *B. subtilis*. The high producing levels of foreign proteins have proved the high efficiency and universality of these two plasmids and stated that they were capable of expressing many other proteins. Furthermore, the practicability of pUC980-2 in industrial applications were strongly illustrated by the stable expressions of heterogenous proteins with pUC980-2 in larger scale fermentations.

As for the relatively lower expressions of PulA11 than the other two proteins, the result indicated that the foreign gene itself also make important effect in the final expression level. Meanwhile, the expression levels of protein PulA11 with plasmid pUC980-2 in *B. subtilis* was still promoted to a certain extent compared with our previous studies in *E. coli* expression system [43–45]. These improvements further confirmed the availability of pUC980-2 in production of heterogenous proteins.

Many *E. coli*–*B. subtilis* shuttle plasmids have been constructed and employed in heterogenous productions mainly based on natural plasmids such as pBS72, pUB110 and pC194 in previous studies [16, 46, 47]. Some of them are inducible, such as pHCMC05 and pHT43 derived from pBS72, which were seldom considerable in economy because of the expensive inducers [48]. There were also some constitutive plasmids originated from pUB110, e.g. pBNS2 and pBSG, which have shown potentials in foreign expressions [49, 50]. However, further developments of these shuttle plasmids were restricted by the lack of systematical analysis about plasmids constructions. In this context, the analyses carried out in this work may have provided some conducts in shuttle plasmids manipulations in the *B. subtilis* expression system. According to our knowledge, it is the first time to classify manipulations with *ori*-insertions to pUB110-derived plasmid in this work.

## Conclusion

In this work, some novel rules about the constructions of *E. coli*-*B. subtilis* shuttle plasmids based on pWB980 were concluded: (1) The deletion of *bleoR* gene improved the plasmid copying of pWB980; (2) The *ori* insertion was generally beneficial to plasmid replication in *B. subtilis*, while its impacts on plasmids segregation stabilities differed with insertion sites; (3) The site upstream the membrane binding region BA3-1 was the optimum position for plasmids manipulations with improvements both in copy number and stability. Based on these construction rules, two plasmids pUC980-1 and pUC980-2 with high copy numbers and high stabilities were selected out and performed well in tested protein productions. The highest production levels in *B. subtilis* of pectate lyase PelN and alkaline protease Spro1 were achieved by using plasmid pUC980-2 as expressing vector through 10 L fermentations. Plasmid pUC980-2 was proved to be capable and valuable in further industrial productions by these promising results.

## Methods

### Bacterial strains, plasmids and materials

The bacterial strains and plasmids used in this study are listed in Additional file 2: Table S1. *E. coli* DH5α was used in plasmids construction and amplification. *B. sutilis* 168 were used in the determinations of plasmids properties. *B. subtilis* WB600 was employed in gene expressions. The sequences of the alkaline pectate lyase from *Paenibacillus* sp. 0602 (*pelN*, GenBank: KC351190.1), the alkaline protease gene *spro1* from alkaliphilic *Bacillus* sp. 221 (*spro1*, Sequence ID: D13157.1) and the pullulanase gene *pulA11* from *Anoxybacillus* sp. LM18-11 (GeneBnak ID: HQ844266.1) were deposited in NCBI. The restriction endonucleases and DNA polymerase were commercially supplied by Thermo Fisher Scientific Co., Ltd. All other enzymes, chemicals and reagents were purchased from TaKaRa Biotechnology (Dalian, China) Co., Ltd.

### Cultivation conditions

All cells were routinely grown at 37 °C and 200 rpm in Luria–Bertani (LB) medium or fermentation medium. LB medium (1 L) consisted of tryptone 10 g, yeast extract 5 g and NaCl 5 g. 1 L of fermentation medium consisted of tryptone 16 g, soluble starch 37 g, CaCl_2_ 1.5 g, NaCl 2 g, MgSO_4_·7H_2_O 0.2 g, KH_2_PO_4_ 1 g, FeSO_4_·7H_2_O 0.05 g and (NH_4_)_2_SO_4_ 1.5 g. Different antibiotics (50 μg/mL kanamycin and 25 μg/mL kanamycin) were added in the medium for the relevant recombinant strains.

*B. subtilis* WB600 strains containing recombinant plasmids were cultured in Shake flask (SHUNIU, GG-17, Sichuan SHUBO Co., LTD, China) or in a 10-L fermenter (5BG, bxbio, China) to produce foreign proteins. The shake-flask culturing was performed as followed. A 5 mL aliquot of LB medium supplemented with 25 μg/mL kanamycin was inoculated with a frozen glycerol stock of recombinant strain (20 μL), and then incubated for up to 14 h at 37 °C and 200 rpm in a rotary shaker (ZQWY-200G, Shanghai Zhichu Instruments Co., Ltd., China). An aliquot of this preculture (0.5 mL) was transferred into a 250 mL shake-flask that was loaded with 50 mL of LB medium supplemented with 25 μg/mL kanamycin, which was then shaken in the rotary shaker (200 rpm) at 37 °C. After culturing for 48 h, samples of each culture were collected and analyzed for enzyme activities. The culture was harvested by centrifugation at 12,000×*g* for 10 min at 4 °C to obtain the culture supernatant. Enzyme activities in the supernatant were determined with corresponding assays. Then the culture supernatant was analyzed with SDS-PAGE. The percentages of the produced extracellular proteins were calculated by a software Gel-Pro analyzer 4.0 (Media Cybernetics, CA, USA).

Fermentation of the recombinant *B. subtilis* WB600 strain was performed in a 10-L fementer. The seed culture was prepared as described in the above section and then used to inoculate an initial batch of fermentation medium supplemented with 25 μg/mL kanamycin. The biomass and enzyme activities were analyzed at designated time intervals. The cell density was determined by measuring the OD_600nm_ with a UV-1800/PC spectrophotometer (Epoch 2, BioTek, USA). To determine the wet cell weight (WCW), a 2-mL sample of the culture broth were centrifuged at 12,000×*g* for 10 min at 4 °C. The resulting pellet was collected and weighed. The enzyme activities in the culture supernatant were further determined with corresponding methods. The whole fermentation process was stopped and finished when continuous reductions of biomass and enzyme activities were observed.

### Shuttle plasmids construction

Construction of shuttle plasmids is schematically presented in Fig. 1. All plasmids were constructed through the method of MEGAWHOP [51]. All of the specific primers used for PCR amplification were synthesized by the Beijing Genomics Institute (BGI) and listed in Table 1 and the isolation and manipulation of recombinant DNA were performed according to the previously published protocol [52]. 1.5 mL of culture was poured into a 2 mL EP tube and centrifuged at 12,000×*g* for 10 min at 4 °C. The supernatant was discarded and the bacterial pellet was collected. The bacterial samples were treated with solution I (100 mL of solution I consisted of 1 M pH 8.0 Tris–HCl, 2.5 mL; 0.5 M EDTA 2 mL; ddH_2_O 91 mL; 20% sterile glucose 4.5 mL), solution II (100 mL of solution II consisted of 10% SDS 50 mL; 2 M NaOH 50 mL), solution III (500 mL of solution III consisted of KAc 147 g; HAc 57.5 mL, ddH_2_O was added to 500 mL), mixture of phenol: chloroform (volume ration 1:1) and 70% ethanol successively according to the protocol. The DNA sample was dissolved with 80 μL ddH_2_O and stored in − 20 °C for further experiments.

**Table 1:** Primers used in this study

Primers	Sequence (5′-3′)
DB-1	AATCTATTATTAATCTGTTCAGCAATC
DB-2	TCTCACGCATAAAATCCCC
P1-S	GGGGATTTTATGCGTGAGACTGTCAGACCAAGTTTACTCAT
P1-A	GATTGCTGAACAGATTAATAATAGATTAGCGGTATCAGCTCACTC
P2-S	GATTGCTGAACAGATTAATAATAGATTCTGTCAGACCAAGTTTACTCAT
P2-A	GGGGATTTTATGCGTGAGAAGCGGTATCAGCTCACTC
P3-S	ATTGAACATACGGTTGATTTAATAACTGACTGTCAGACCAAGTTTACTCAT
P3-A	GCTTTAGCAAGAGGGTGATGTTTGAGCGGTATCAGCTCACTC
P4-S	GCTTTAGCAAGAGGGTGATGTTTGCTGTCAGACCAAGTTTACTCAT
P4-A	GAACATACGGTTGATTTAATAACTGAAGCGGTATCAGCTCACTC
P5-S	CAAGAAAAACACGATTTAGAACCCTGTCAGACCAAGTTTACTCAT
P5-A	GAGTTAGTTCAAATTCGTTCTTTTTAAGCGGTATCAGCTCACTC
P6-S	GAGTTAGTTCAAATTCGTTCTTTTTACTGTCAGACCAAGTTTACTCAT
P6-A	CAAGAAAAACACGATTTAGAACCAGCGGTATCAGCTCACTC
P7-S	TGCTTAGGAAGACGAGTTATTAATACTGTCAGACCAAGTTTACTCAT
P7-A	GAATATTTGGAGAGCACCGTTCTTATTCAGCAGCGGTATCAGCTCACTC
P8-S	GAATATTTGGAGAGCACCGTTCTTATTCAGCCTGTCAGACCAAGTTTACTCAT
P8-A	TGCTTAGGAAGACGAGTTATTAATAAGCGGTATCAGCTCACTC
62-S	CATAAAAAAGGAGACATGAACGATGGCGGGCAATGCAGATTAC
62-A	CCCCGGGTACCGAGCTCGATTAATAGCTCGTCTTCAGCCAGTTGTCC
spro-S	GCGCAACTCAAGCTTTTGCCATGAAGCTTAAGAAACCGTTG
spro-A	CCCGGGTACCGAGCTCGATTAGCGTGTTGCCGCTTC
pulA-S	GAGGCGCAACTCAAGCTTTTGCCCCCCCAAAACAACAGTCGTTTGAAG
pulA-A	GATCCCCGGGTACCGAGCTCGATCAACATTGAATTAATACCCACGCAC
kanS	CGGATATTGAGATGATGTGTGTCATGTC
kanA	GACCATGTGTAAGCGGCCAATC
dnaNS	GCACTTGCCGCAGATTGA
dnaNA	AATGCAAGACGGTGGCTATC

According to the map obtained after sequencing, the 169 bp fragment of the membrane-binding region BA3 originating from pUB110 was deleted during construction of pWB980. In order to be mentioned conveniently, the remaining 346 bp fragment of BA3 was named BA3-1 in this work as shown in Fig. 1. pWB980 is consist of five parts: BA3-1, P43 promoter cohered with a signal-peptide sequence, the replicase-coding gene *rep* in *B. subtilis,* the kanamycin-resistance gene *kanR* and the bleomycin gene *bleoR* gene (Fig. 1). In this work, the *bleoR* was deleted with primers DB-1/DB-2. The plasmid replication origin *ori* (888 bp) from pUC19 was inserted at the four sites up- and down-stream BA3-1 region and *rep* gene in pWB980-DB (3388 bp) separately. With primer pairs P1-S/P1-A and P2-S/P2-A, *ori* integrated into the site (659 bp) upstream the BA3-1 with forward and reverse directions. The recombinant plasmids were named as pUC980-1 and pUC980-2 respectively. At the site (169 bp) downstream the BA3-1, primers P3-S/P3-A and P4-S/P4-A were used in *ori*-insertions, resulting plasmids pUC980-3 and pUC980-4 in forward and reverse directions. Upstream the *rep* gene, the *ori* was inserted at the 3118 site using primers P5-S/P5-A and P6-S/P6-A. The recombinant plasmids were named as pUC980-5 and pUC980-6 with forward and reverse *ori*-insertions respectively. *Ori* was put into the 1722 site downstream the *rep* by using primers P7-S/P7-A and P8-S/P8-A. The plasmid with forward *ori* insertion was pUC980-7 and the other one was pUC980-8.

With primer pair 62-S/62-A, the alkaline pectate lyase gene *pelN* was inserted into the sites after the P43 promoter of plasmids pWB980, pWB980-DB and all pUC980-serial plasmids except pUC980-8. The recombinant plasmids were pWB980-*pelN*, pWB980-DB-*pelN*, pUC980-1-*pelN,* pUC980-2-*pelN*, pUC980-3-*pelN,* pUC980-4-*pelN*, pUC980-5-*pelN*, pUC980-6-*pelN* and pUC980-7-*pelN* correspondently.

### Determination of plasmid copy number by quantitative real time PCR (qPCR)

The quantitative real-time PCR (qPCR) was performed using iQ™ SYBR^®^ Green Supermix (Bio-Rad) as described by Turgeon [53]. The *dnaN* and *kanR* genes were chosen as a single copy reference gene on the *B. subtilis* chromosome and the test gene in plasmids correspondingly [54–56]. Specific primer pairs dnaNS/dnaNA and kanS/kanA (Table 1) were designed to generate products of approximately 150 bp. The standard curves were prepared based on the pMD-18-T vector. The PCR mixtures (total volume 20 μL) contained 10 μL 2xTakara SYBR Green Real-Time PCR Master Mix, 1.5 μL forward and reverse primers (10 μM) and 1 μL diluted (10^−1^ to 10^−4^) sample DNA. The reaction condition was as followed: 95 °C for 10 min, 45 cycles of 95 °C for 10 s, 62 °C for 20 s, and 72 °C for 30 s, followed by a gradient temperature from 55 °C to 95 °C. The calculated mean of the *kanR* gene in each compared plasmid was divided by the *dnaN* gene (single-copy reference), resulting the copy number. All experiments were performed with three independent biological replicates.

### Plasmid stability assay

Plasmid segregational stability in *B. subtilis* 168 was detected according to the method of Bron and Luxen [39] with minor modifications. Single colonies on kanamycin selective (25 μg/mL) LB agar plate were used to inoculate 5 mL kanamycin selective (25 μg/mL) LB medium. After 24 h culturing at 37 °C, the cultures were diluted 1:1000 in fresh LB media without antibiotics and incubated at 37 °C. This iterative subculturing process was repeated every 24 h for 30 consecutive days. Samples taken at appropriate intervals were checked for the fraction of plasmid-containing cells by replica plating on LB agar plates containing 25 μg/mL kanamycin.

$$ {\text{The plasmid segregational stability }}\left( {\text{\% }} \right) = \frac{\text{N}}{100}*100\% $$

N: Total amount of colonies on the selectable plate.

Plasmids were extracted from host cells after 30-days continuously culturing and detected the structural stability by EcoRI/HindIII enzyme-digestion verifications.

### Alkaline pectate lyase assay

The pectate lyase activity was measured using the method described previously by Wang et al. [57]. 20 μL of the diluted enzyme solutions were added to 400 μL of 0.2% pectin in 50 mmol/L glycine–NaOH buffer (with 50 μmol/L CaCl_2)_ at pH 9.0. The reaction mixture was incubated at 65 °C for 10 min, and the reaction was terminated by adding 580 μL of 30 mmol/L H_3_PO_4_. The product yields were detected using a spectrophotometer at 235 nm (Epoch 2, BioTek, USA). One standard enzyme unit was defined as the yield of 1 μmol unsaturated pectin per minute under the above-mentioned conditions.

### Alkaline protease assay

The alkaline protease activity was measured using the modified method described by Tjalsma et al. [28]. One standard enzyme unit was defined as the as the amount of enzyme that hydrolyzes 1 μg casein minute under the situation of pH 9.0, 50 °C. 250 μL of the diluted enzyme solution were mixed with 250 μL solution containing 2% casein in pH 9.0 buffer. The mixture was incubated at 50 °C for 10 min, and the reaction was terminated by adding 500 μL 0.4 M TCA. The productions were detected using a spectrophotometer (Epoch 2, BioTek, USA) at 280 nm. Casein with concentrations from 0 to 500 μg/mL were used as standard.

### Pullulanase assay

Pullulanase activity was measured using the modified dinitrosalicylic acid (DNS) method [58]. One standard enzyme unit was defined as the amount of enzyme that releases 1 μmol reducing sugar per minute under the assay conditions. Glucose with concentrations from 0 to 400 mg/mL were used as standard. 50 μL of the diluted enzyme solutions were added into the 450 μL reaction mixture containing 5% pullulan solutions and the pH 6.0 buffer at the volume ratio of 1:8. After incubations at pH 6.0, 60 °C for 30 min, 500 μL DNS were used to stop the reactions. The productions were detected using a spectrophotometer (Epoch 2, BioTek, USA) at 540 nm. The amount of reducing sugar released during incubations were tested as the enzyme-index.

### SDS-PAGE

*B. subtilis* WB600 transformant cells harboring recombinant plasmids were cultured in LB medium containing 25 μg/mL kanamycin at 37 °C for 48 h. The cultures (0.5 mL respectively) were centrifuged at 5000 rpm, 4 °C for 10 min and the supernatant solutions were reserved. The cell pellets were re-suspended in 0.5 mL ddH_2_O, followed by sonication to release intracellular content. Extracellular proteins were precipitated with 13% (w/v) TCA for 30 min on ice, then the precipitates which were collected by 10 min centrifugation at 4 °C and 13,000×*g*, were carefully washed with ice-cold acetone and dried under vacuum. The collective proteins were dissolved in 20 μL urea (6 M) and mixed with appropriate volume of 4 × SDS loading buffer. Samples were then heated in boiling water bath for 10 min and centrifuged at 13,000×*g* for 10 s. 12 μL of the samples were loaded onto 10% SDS-PAGE and run at 20 mA for around 1 h. Gel was stained using Coomassie brilliant blue dye.

## Supplementary information

**Additional file 1: Figure S1.** 10 L fermentations of Spro1 and PulA11. Alkaline protease Spro1 and pullulanase PulA11 were further produced in 10 L fermentator in *B. subtilis* WB600 with plasmid pUC980-2. The cell growth curves and activities were tested every 6 h. **A.** During the fermentation of Spro1, cells reached at the stationary phase at 30 h and the highest activity was 21537 U/mL at 60 h. **B.** the growth curve of the WB600 (pUC980-2-*pulA11*) was similar to the PelN and Spro1 productions. The cells went into the stationary phase at 30 h and the highest production was 504 U/mL at 48 h.

**Additional file 2: Table S1.** Strains and plasmids used in this study.

## Data Availability

We declared that materials described in the manuscript, including all relevant raw data, will be freely available to any scientist wishing to use them for non-commercial purposes, without breaching participant confidentiality.

## Abbreviations

SDS-PAGE: Sodium dodecyl sulfate–polyacrylamide gel electrophoresis
BGI: The Beijing Genomics Institute
qPCR: The quantitative real-time PCR
