# Supplementary figures and images for: High copy number and highly stable Escherichia coli–Bacillus subtilis shuttle plasmids based on pWB980
Source: Microb Cell Fact. 2020 Feb 7;19:25. doi: 10.1186/s12934-020-1296-5 (PMC7006159; doi:10.1186/s12934-020-1296-5)

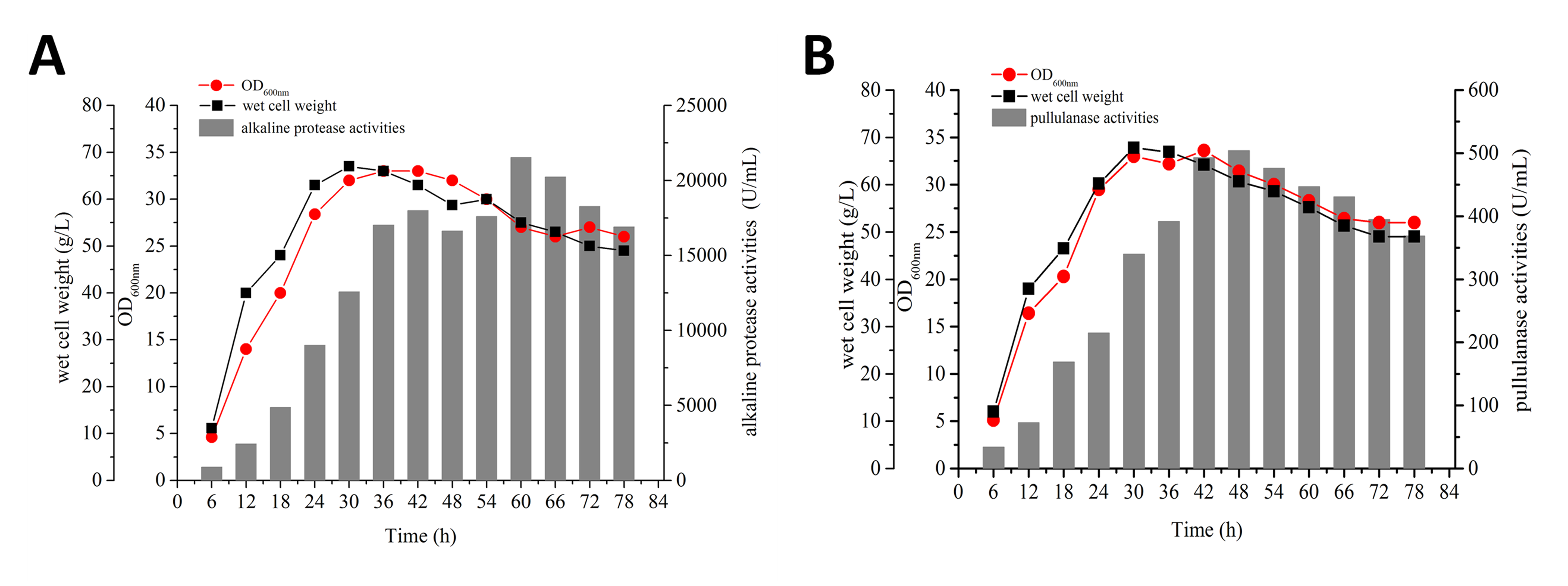

Supplement: Supplementary file 1 — Additional file 1: Figure S1. 10 L fermentations of Spro1 and PulA11. Alkaline protease Spro1 and pullulanase PulA11 were further produced in 10 L fermentator in B. subtilis WB600 with plasmid pUC980-2. The cell growth curves and activities were tested every 6 h. A. During the fermentation of Spro1, cells reached at the stationary phase at 30 h and the highest activity was 21537 U/mL at 60 h. B. the growth curve of the WB600 (pUC980-2-pulA11) was similar to the PelN and Spro1 productions. The cells went into the stationary phase at 30 h and the highest production was 504 U/mL at 48 h. [file 12934_2020_1296_MOESM1_ESM.png]
