# Supplementary material for: High copy number and highly stable Escherichia coli–Bacillus subtilis shuttle plasmids based on pWB980
Source: Microb Cell Fact. 2020 Feb 7;19:25. doi: 10.1186/s12934-020-1296-5 (PMC7006159; doi:10.1186/s12934-020-1296-5)
Supplement: Supplementary file 2 — Additional file 2: Table S1. Strains and plasmids used in this study. [file 12934_2020_1296_MOESM2_ESM.docx]

**Table S1 Strains and plasmids used in this study**

| **strains and plasmids** | **characteristics** | **reference** |
| --- | --- | --- |
| **strains** | | |
| *E. coli* DH5α | F-,φ80d*lacZ*△M15, △(*lacZYA-argF*)U169, *deoR*, *recA*1, *endA*1, *hsdR*17(rk-,mk+ ), *pho*A, *supE*44, λ-, *thi*-1, *gyrA*96, *relA*1 | Takara |
| *B. subtilis* 168^a^ | *trp*C2 | ATCC |
| *B. subtilis* WB600 | *apr*, *npr*A, *epr*, *bpf*, *mpr*, *npr*B, *trp*C2 | Our lab. preservation |
| **plasmids** | | |
| pMD-18-T | Standard vector for plasmid copy number determination, Amp^r^ | Takara |
| pUB110 | Kan^r^, *mob* region, membrane biding region BA3 | Our lab. preservation |
| pWB980 | pUB110 derivative, destroyed *mob* region, Kan^r^, Bleo^r^, P43 promoter, part BA3 region(BA3-1) | Our lab. Preservation |
| pWB980-DB | pWB980 derivate, *bleoR* was deleted, Kan^r^, P43 promoter, BA3-1 region | this work |
| pUC980-1 | *B.subtilis-E.coli* shuttle expression vector, Kan^r^, P43 promoter, *ori* was inserted between BA3-1 region and *kanR* gene in homodromous direction | this work |
| pUC980-2 | *B.subtilis-E.coli* shuttle expression vector, Kan^r^, P43 promoter, *ori* was inserted between BA3-1 region and *kanR* gene in reversed direction | this work |
| pUC980-3 | *B.subtilis-E.coli* shuttle expression vector, Kan^r^, P43 promoter, *ori* region was inserted between BA3-1 region and P43 promoter in forward direction | this work |
| pUC980-4 | *B.subtilis-E.coli* shuttle expression vector, Kan^r^, P43 promoter, *ori* region was inserted between BA3-1 region and P43 promoter in reversed direction | this work |
| pUC980-5 | *B.subtilis-E.coli* shuttle expression vector, Kan^r^, P43 promoter, *ori* was inserted between P43 promoter and *rep* in homodromous direction | this work |
| pUC980-6 | *B.subtilis-E.coli* shuttle expression vector, Kan^r^, P43 promoter, *ori* was inserted between P43 promoter and *rep* in reverse direction | this work |
| pUC980-7 | *B.subtilis-E.coli* shuttle expression vector, Kan^r^, P43 promoter, *ori* was inserted between *rep* and *kanR* in forward direction | this work |
| pUC980-8 | *B.subtilis-E.coli* shuttle expression vector, Kan^r^, P43 promoter, *ori* was inserted between *rep* and *kanR* in reverse direction | this work |
| pWB980-*pel*N | Kan^r^, Bleo^r^, P43 promoter, signal peptide *sacB*, control plasmid in Alkaline pectate lyase *pel*N expression in *B. subtilis* with copy number in C3 and segregation stability of S1 | this work |
| pWB980-DB-*pel*N | Kan^r^, P43 promoter, signal peptide *sacB*, *pel*N expression plasmid in *B. subtilis* with copy number in C2 and segregation stability of S1 | this work |
| pUC980-1- *pel*N | Kan^r^, P43 promoter, signal peptide *sacB*, with both high copy number and segregation stability, *pel*N expression plasmid in *B. subtilis* with copy number in C1 and segregation stability of S1 | this work |
| pUC980-2-*pel*N | Kan^r^, P43 promoter, signal peptide *sacB*, with both high copy number and segregation stability, *pel*N expression plasmid in *B. subtilis* with copy number in C3 and segregation stability of S1 | this work |
| pUC980-3-*pel*N | Kan^r^, P43 promoter, signal peptide *sacB*, *pel*N expression plasmid in *B. subtilis* with copy number in C2 and segregation stability in S3 | this work |
| pUC980-4-*pel*N | Kan^r^, P43 promoter, signal peptide *sacB*, *pel*N expression plasmid in *B. subtilis* with copy number in C2 and segregation stability in S2 | this work |
| pUC980-5- *pel*N | Kan^r^, P43 promoter, signal peptide *sacB*, control plasmid with low copy number and high segregation stability, *pel*N expression plasmid in *B. subtilis* with copy number in C2 and segregation stability in S1 | this work |
| pUC980-6-*pel*N | Kan^r^, P43 promoter, signal peptide *sacB*, control plasmid with high copy number and low segregation stability, *pel*N expression plasmid in *B. subtilis* with copy number in C1 and segregation stability in S3 | this work |
| pUC980-7-*pel*N | Kan^r^, P43 promoter, signal peptide *sacB*, control plasmid with high copy number and low segregation stability, *pel*N expression plasmid in *B. subtilis* with copy number in C3 and segregation stability in S3 | this work |
| pWB980-*spro1* | Kan^r^, Bleo^r^, P43 promoter, control plasmid in alkaline protease *spro1* expression in *B. subtilis* with copy number in C3 and segregation stability of S1 | this work |
| pWB980-DB- *spro1* | Kan^r^, P43 promoter, control plasmid in alkaline protease *spro1* expression in *B. subtilis* with copy number in C2 and segregation stability of S1 | this work |
| pUC980-1- *spro1* | Kan^r^, P43 promoter, with both high copy number and segregation stability, *spro1* expression plasmid in *B. subtilis* with copy number in C1 and segregation stability of S1 | this work |
| pUC980-2- *spro1* | Kan^r^, P43 promoter, with both high copy number and segregation stability, *spro1* expression plasmid in *B. subtilis* with copy number in C3 and segregation stability of S1 | this work |
| pWB980-*pulA11* | Kan^r^, Bleo^r^, P43 promoter, signal peptide *sacB*, control plasmid in pullulanase gene *pulA11* expression in *B. subtilis* with copy number in C3 and segregation stability of S1 | this work |
| pWB980-DB- *pulA11* | Kan^r^, P43 promoter, signal peptide *sacB*, *pulA11* expression plasmid in *B. subtilis* with copy number in C2 and segregation stability of S1 | this work |
| pUC980-1- *pulA11* | Kan^r^, P43 promoter, signal peptide *sacB*, with both high copy number and segregation stability, *pulA11* expression plasmid in *B. subtilis* with copy number in C1 and segregation stability of S1 | this work |
| pUC980-2- *pulA11* | Kan^r^, P43 promoter, signal peptide *sacB*, with both high copy number and segregation stability, *pulA11* expression plasmid in *B. subtilis* with copy number in C3 and segregation stability of S1 | this work |

^a^ *Bacillus subtilis* 168 (ATCC23857) was provided by the American Type Culture Collection (ATCC).
